# Magnetic Beads of Zero Valent Iron Doped Polyethersolfun Developed for Removal of Arsenic from Apatite-Soil Treated Water

**DOI:** 10.3390/ijerph191912697

**Published:** 2022-10-04

**Authors:** Roya Noorbakhsh, Mohammad Kazem Koohi, Jalal Hassan, Anosheh Rahmani, Hamid Rashidi Nodeh, Shahabaldin Rezania

**Affiliations:** 1 Department of Comparative Bioscience, Faculty of Veterinary Medicine, University of Tehran, Tehran 1419963111, Iran; 2 Food Technology and Agricultural Products Research Center, Standard Research Institute (SRI), Karaj 3174734563, Iran; 3 Department of Environment and Energy, Sejong University, Seoul 05006, Korea

**Keywords:** polyethersulfone beads, zero valent iron, apatite-soil, arsenic, adsorption, kinetic

## Abstract

The drop immerses calcium chloride aqueous solution was utilized to prepare the zero valent iron-doped polyethersulfone beads (PES/ZVI) for the efficient removal of arsenic from apatite-soil treated waters. The proposed beads can assist in promoting uptake efficiency by hindering ZVI agglomeration due to a high porosity and different active sites. The PES/ZVI beads were characterized by Fourier transform infrared spectroscopy (FTIR), scanning electron microscopy (SEM), thermogravimetric analysis (TGA), and vibrating sample magnetism (VSM). The main objective of this study was to investigate the function of new PES/ZVI beads with an increased removal efficiency for the remediation of arsenic ions from the apatite-soil treated waters. A maximum adsorption removal of 82.39% was achieved when the experiment was performed with 80 mg of adsorbent for a contact time of 180 min. Based on the results, a removal efficiency >90% was obtained after 300 min of shaking time with an arsenic concentration of 20 mg·L^−1^. The experimental process was fitted with the Langmuir model due to the high R^2^ (0.99) value compared to the Freundlich model (0.91) with an adsorption capacity of 41.32 mg·g^−1^. The adsorption process speed was limited by pseudo-second-order (R^2^ = 0.999) and the adsorption mechanism nature was endothermic and physical.

## 1. Introduction

Apatite is a mineral soil, and the main source of phosphate fertilizer in different countries. Apatite-soil contains a high level of arsenic, which is easy to enter into fertilizer, causing the contamination of farmland and crops. There are many health risks from consuming arsenic, yet obtaining these risks merely from the inhalation of soil enriched with arsenic is well-reported. Hence, this can create a cascade of problems in human ecosystems and lead to physiological and neurological disorders. Owing to the immense distribution of arsenic and its toxicity, it is considered a carcinogenic and geogenic metal across the world. Arsenic species in the environment have two forms: an organic form (monomethyl arsenate and dimethyl arsenate) and an inorganic form (arsenite and arsenate). Hence, the main forms of arsenic in the environment are inorganic arsenate (As V) and arsenite (As III) [1,2]. The other source of arsenic in soil/water is due to the unprecedented increase in industry-related activities and agricultural demand such as mining, swine feed additives, pesticides, herbicides, wood treatment agents, electronic manufacturing, pharmaceuticals, dyes, cosmetics, and coal combustion [3,4,5]. Considering the wide distribution of arsenic species and their cancerous and non-cancerous impacts, it is urgent to control and monitor the concentration of arsenic species in the environment for the sake of public health. Therefore, the World Health Organization (WHO) implemented a strict permission level for the existence of arsenic in soil (50 mg·kg^−1^) and potable water (10 µg·L^−1^) [5,6].

The contaminated soil by arsenic is less tangible than water and air, therefore it can threaten the health of humans from various exposure pathways. In this regard, the treatment of soil is essential to guarantee the health of humans and the ecosystem. To reduce the health risks of As-contaminated soil, researchers have developed a wide variety of approaches to remove arsenic from soil and reduce its biotoxicity. The remediation of arsenic from soil and water can be classified as biological oxidation, electrokinetics, phytoremediation, coagulation-flocculation, and solidification/stabilization [7,8]. However, the adsorption process can also utilize to remove arsenic from the water of treated soil.

Among diverse approaches for arsenic remediation from aqueous media, the adsorption technique is the most promising approach and has many advantages such as a low cost, high efficiency, and ease of operation [9]. The application of iron-based adsorbents is well-documented, owing to the acceptable extraction efficiency for arsenic species from environmental samples [10]. Moreover, granular ferric hydroxide and zero-valent iron (ZVI) have been produced as commercial adsorbents to uptake arsenic [11]. Although, there are some disadvantages to ZVI adsorbents such as its fast oxidation in aqueous media, due to the small size of ZVI, it can easily be agglomerated via the van der Waals attractive, which leads to bigger particles, decreasing the reactivity [12,13]. To overcome these issues and improve the stability and reusability of ZVI, the incorporation of ZVI into polymer support, such as polyethersulfone (PES), will be helpful [14]. Then, in this study the microbeads based on PES/ZVI were fabricated for the efficient removal of arsenic ions.

The object of the present study is to eliminate arsenic from apatite-soil (general formula of A_10_(XO_4_)_6_(F, OH, Cl)_2_, (X = P^+5^, As^+5^, V^+5^)), since apatite-soil contains a large amount of arsenic [15]. The removal process was conducted in a two-step process. Initially, the soil samples were treated by using the acidic water (pH ~ 3), and then the released free arsenic ions in the aqueous solution were adsorbed onto magnetic microbeads (PES/ZVI). Moreover, the incorporation of ZVI into PES beads prevents the agglomeration and oxidation of iron (Fe^0^ or ZVI) and improves the synergic effects, as well as increases the adsorption efficiency. Hence, the proposed method is prevented to discharge the arsenic ions into the environment after the treatment of the apatite-soil.

## 2. Materials and Methods

### 2.1. Chemical and Reagents

Polyethersulfone (PES) with 75,000 MW and an industrial grade was purchased from BASF (Ludwigshafen, Germany). Dimethylformamide (DMF), ferrous (III)chloride hexahydrate, sodium boron hydride, methanol, hydrochloric acid, and sodium hydroxide were purchased from Merck chemical groups (Darmstadt, Germany).

### 2.2. Preparation of Apatite-Soil

Apatite-soil was obtained from a mine in the central area of Iran (Bafgh, Yazd) with hot and dry weather and a desert geographical area. The apatite was blended with an excavator in the mine. Then, this mixture was screened by a 350 μm sieve. After sieving, the sample was dried at 50 °C for 24 h and utilized for the soil treatment test under various conditions and different adsorbents.

### 2.3. Treatment of Apatite-Soil Experiment

Apatite-soils were chosen with an arsenic concentration in the range of 72.32–170.59 mg·L^−1^ (Table 1). Ten g of each soil was dissolved in 100 mL of double-distilled water (DDW) and sonicated for 1 h to obtain the disperse solution, and it was then divided to three parts and placed in different solutions with different pH values (3, 6, and 10). These samples were shaken at room temperature for 24 h. Then, the samples were centrifuged and the concentration of the released arsenic in the aqueous phase was measured with flame atomic absorption (FAAS), as listed in Table 1.

### 2.4. Beads Charactrization

Elemental composition and surface morphology were studied using a Field Emission Scanning electron microscope (EM8000 KYKY) equipped with an energy dispersive accessory (SEM/EDX). IR spectra were recorded in the range of 400–4000 cm^−1^ using a Bruker ATR-FTIR spectrometer (Bremen, Germany). Thermogravimetric analysis (TGA) was utilized for the evaluation of the thermal resistance of the adsorbent. For the determination of the magnetic properties of the adsorbent, VSM was applied.

### 2.5. Synthesis of PES/ZVI Microbeaads

The synthesis of magnetic zero-valent iron particles (ZVI) from ferrous was carried out by the precipitation method [11]. A total of 2.4 g of ferrous chloride (Fe^3+^) was dissolved in 50 mL of DDW, separately, and 15 mL of 1.5 M of NaBH_4_ was added dropwise, with stirring taking place at 50 °C. The black precipitate was separated magnetically and washed three times with DDW and ethanol and dried at 80 °C for 90 min (see Equation (1)).

**Figure 1:**
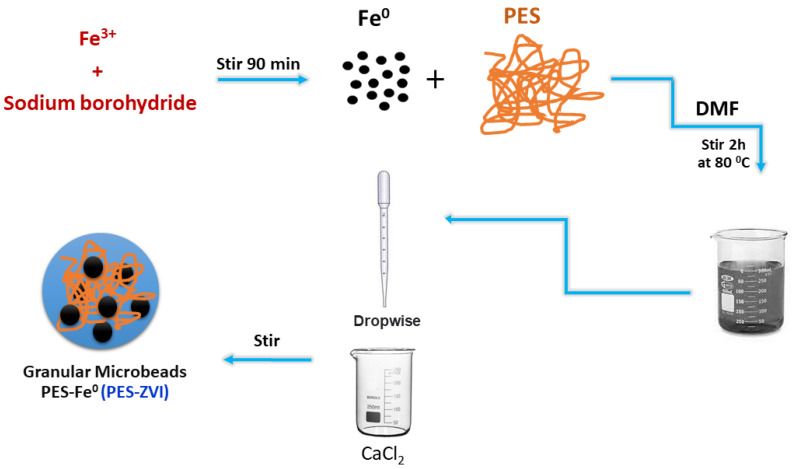
Schematic route for the synthesis of PES-Fe^0^ beads (PES-ZVI).

To prepare the magnetic beads based on ZVI over polyethersulfone (PES) as shown in Figure 1, 0.5 g of ZVI with 1 g of PES polymer were dissolved in 50 mL of DMF and heated up to 80 °C for 120 min of stirring. Then, the mixture was added into DDW dropwise to get the beads and it was stabilized with a 0.1 M solution of calcium chloride for 120 min. The obtained functionalized magnetic beads (PES/ZVI) were separated by applying an external magnet, then they were properly washed with DDW and dried at room temperature.

### 2.6. Adsorption/Removal Procedure

After the acidic washing of the apatite soli (GTSP), the aqueous phase was collected and used for the adsorption experiment. The GTSP-apatite sample with a concentration of 163 mg·L^−1^ was selected for the adsorption process due to it having the highest value of arsenic. The removal process was conducted in a batch mode (diluted GTSP-apatite’s water to different concentrations in the ranges of 10–163 mg·L^−1^) in the presence of PES/ZVI beads as the adsorbents. The concentrations > 163 mg·L^−1^ to 1000 mg·L^−1^ were obtained by spiking the standard arsenic solution onto the treated water samples. The effective parameters were investigated as the effect of the pH (2–10), the mass of the beads (5–100 mg), and the adsorption time (5–300 min) with diluted GTSP-apatite treated water (20 mg·L^−1^). After each experiment, the concentrations of arsenic ions in the aqueous media (GTSP treated water) were measured by atomic absorption spectroscopy (FAAS). The percentage of arsenic removed and the adsorbent capacity was achieved using Equations (1) and (2), respectively:

(1) $$R\%=\frac{\left(C_{0}-C_{e}\right)}{C_{0}}\times 100$$

(2) $$\mathrm{Qe}=\frac{\left(C_{0}-C_{e}\right)\times V}{m}$$

where *C*_0_ and *C*_e_ are the initial and equilibrium concentrations of metal ions in the solution (mg·L^−1^), *V* is the volume of the solution (mL), and *m* is the mass of the adsorbent (mg).

## 3. Results and Discussions

### 3.1. Characterization

#### 3.1.1. FTIR

**Figure 2:**
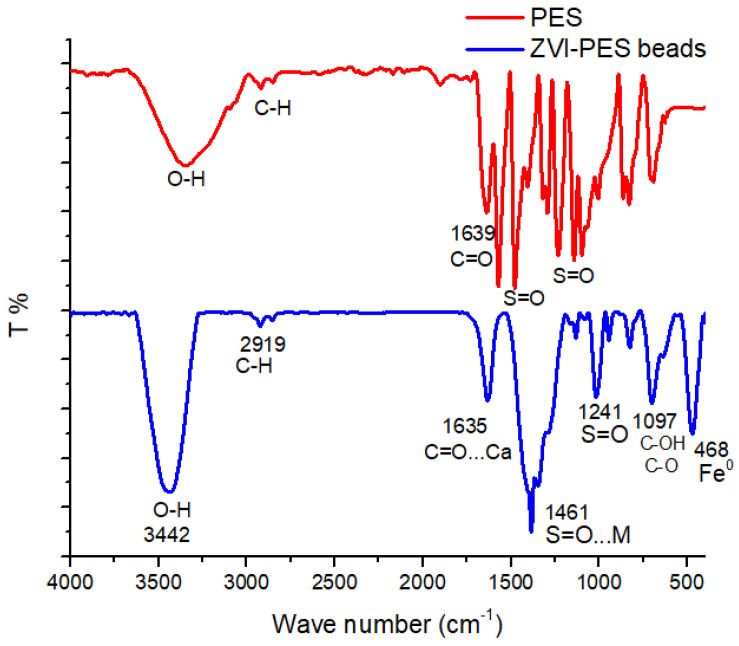
FTIR spectra of PES and PES/ZVI beads.

The synthesis of zero-valent iron on the polyethersulfone was confirmed by recording FTIR. Figure 2 displays the FTIR spectra of plain PES and PES-ZVI beads. In the spectrum of the PES, stretching peaks of C=O can be observed at 1639 cm^−1^ and C-H at 2930 cm^−1^. The sharp peak at 1578 cm^−1^ was ascribed to the stretching vibration of the C=C skeleton in the aromatic ring. The intensive peaks at 1461 cm^−1^ and 1241 cm^−1^ were associated with S=O in the backbone of the polymer. The broad peak at 3000–3600 cm^−1^ was associated with the adsorbed water and stretching of O-H...O. The appearance and disappearance of some IR band for the PES/ZVI confirmed the successful incorporation of ZVI on the surface of the PES beads. In addition to the previous peaks, a stretching vibration at 468 cm^−1^ corresponded to the metal-oxide group (Fe-O) [14,16].

#### 3.1.2. TGA

**Figure 3:**
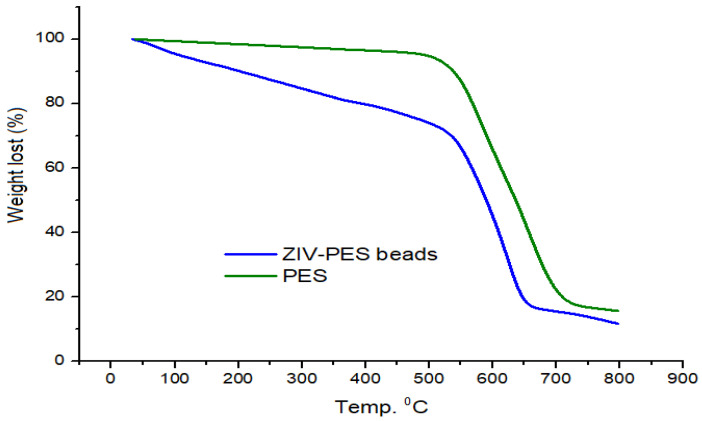
TGA curves of plain PES and PES/ZVI beads.

The thermal stability of PES and PES/ZVI is illustrated in Figure 3. The analysis of the thermogravimetric property was carried out in the range of 23–800 °C with a speed of 10 °C per minute to evaluate the thermal resistance of the adsorbent. According to Figure 3, the thermal resistance of PES was reduced after adding ZVI nanoparticles because of the dispersing of these nanoparticles on the surface of the polymer. This distribution made a disorder on the surface of the PES which reduced the uniformity of the polymer, resulting in a reduction in the thermal resistance of the adsorbent. Although the incorporation of ZVI decreased the thermal stability of the PES, the presence of ZVI improved the adsorption capacity and the easy separation of the beads from the aqueous solution with the assistance of an external magnet.

#### 3.1.3. SEM Microscopy

**Figure 4:**
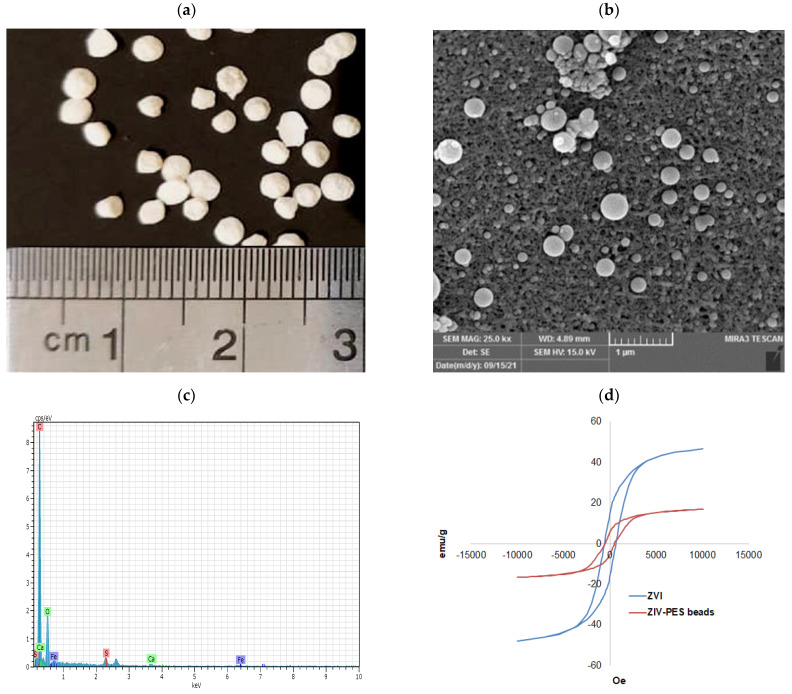
SEM images of (**a**) microbead magnification 5 kx and (**b**) surface of PES/ZVI beads magnification 25 kx. (**c**) EDX spectra of PES/ZVI beads. (**d**) VSM magnetic hysteresis loop for plain ZVI and PES/ZVI beads.

The characterization of the surface of the PES/ZVI beads was measured by a scanning electron microscope (SEM). Figure 4a shows the digital photograph of the PES/ZVI beads with spherical shapes with smooth and uniform surfaces and in the range of 1–3 mm. With increasing the magnification, the SEM images (b) confirmed the distribution of ZVI nanoparticles on the surface of the PES beads. To better understand, the magnifying surface of the adsorbent provides direct visual evidence of the PES/ZVI beads, and sponge-like pores are present with a high dispersity of ZVI on the surface of the PES polymer. These sponge-like pores were interconnected with each other and with the nanometric surface pores. This property could significantly enhance the porosity and surface-to-area ratio, resulting in a high potential to capture arsenic ions [17,18].

Energy dispersive X-ray spectroscopy (EDX) was performed on the PES/ZVI beads to determine the elemental composition. Figure 4c verifies the presence of the main elements on the surface of the PES/ZVI beads, including Fe, S, C, and O, as expected. The weight percentage of these elements was 7.5%, 3%, 55.02%, and 33.95%, respectively. The existence of the elements confirmed the successful synthesis of the PES/ZVI beads for the treatment of arsenic from soil.

#### 3.1.4. VSM

Figure 4d illustrates the magnetic properties of the ZVI nanoparticles and the PES/ZVI beads with VSM magnetization curves. Figure 4 illustrates conductivity in all the magnetic hysteresis, which confirms the superparamagnetic trait. The saturation magnetization values of ZVI and the PES/ZVI beads were achieved at 41 emu/g and 18 emu/g, respectively. This trend followed the previous studies and performs the appropriate magnetic properties for the ZVI and the beads [19,20].

### 3.2. Effect of Parameters on Removal Efficiency

#### 3.2.1. Type of Adsorbent

**Figure 5:**
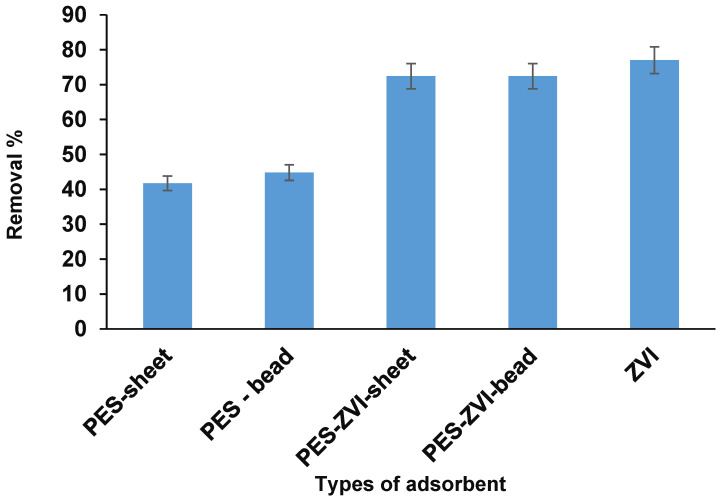
The study of different adsorbents on the uptake of arsenic from apatite-soil treated waters (each material was used 50 mg adsorbent, pH 5, 120 min reaction time, and 20 mg·L^−1^ arsenic).

A comparison of various adsorbents to uptake arsenic from the apatite-soil treated waters was conducted. According to Figure 5, various in-house adsorbents including plain ZVI, PES sheets, ZVI-PES sheets, PES beads, and ZVI-PES beads were used in a similar experimental condition (50 mg of each material, 120 min reaction time, and 20 mg/L arsenic). The plain ZVI particles have a high efficiency in comparison with the others. This trend was resulted from a high reactivity with a standard redox potential and can react with arsenic contaminants easily. While ZVI has some disadvantages for the remediation of arsenic, and employing these nanoparticles independently had some implications, including the agglomeration in water, fast oxidation, high cost, and that fact that it produces pollution that leads to a reduction in its reactivity. Some types of nanoparticles have been considered as toxic substances for a wide distribution in solutions, their application should be aligned with supporting materials that mitigate the leaching of nanoparticles in the solution. In this regard, loading ZVI into the PES beads as a support can reduce the agglomeration, resulting in promoting the efficiency of the adsorbent dramatically and preventing ZVI oxidation [21,22]. Additionally, due to the synergic effects, a lesser amount of ZVI was incorporated into the PES beads.

#### 3.2.2. Solution pH and Proposed Mechanism

**Figure 6:**
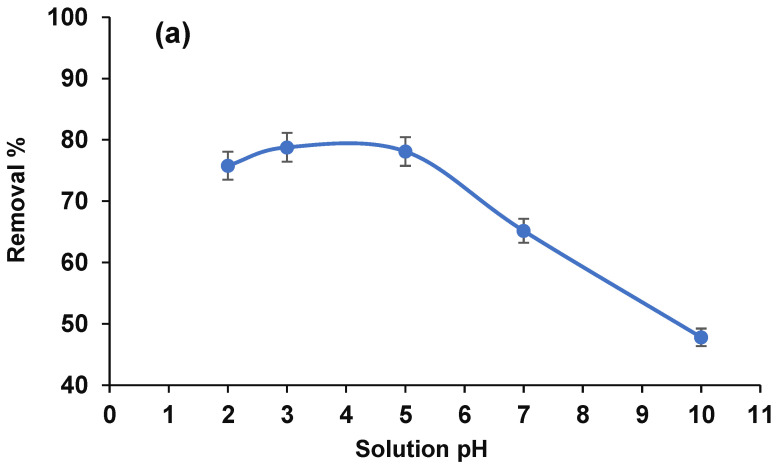

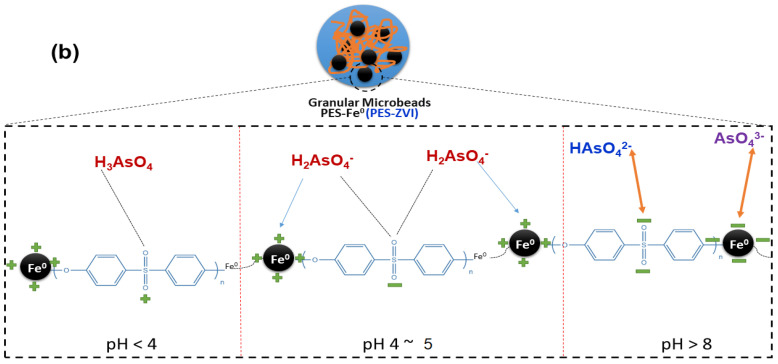
(**a**) The effect of pH on the removal percentage (50 mg adsorbent, 120 min reaction time, and 20 mg·L^−1^ of arsenic) and (**b**) proposed adsorption mechanism at different pH values.

The solution pH is a contributing parameter in the extraction efficiency of arsenic since it can not only impact the metal retention of apatite-soils, but also can change the capability of washing agents to release arsenic from the apatite-soils. The extraction efficiency of arsenic was investigated in the pH range of 2–10 using PES/ZVI beads (Figure 6a). The results present that the high removal efficiency of arsenic metals was generally achieved at an acidic pH (2–4) because, under such circumstances (Figure 6b), the pentavalent form of arsenate or As(V) can be H_2_AsO_4_^-^ or HAsO_4_^2-^ and it had more inclination than arsenite to form during precipitation in the presence of a cation. At a pH value < 4, the protonation and proton competition decrease the adsorption efficiency (Figure 6b). In a higher pH (6–10), the pentavalent form is arsenite (As V), present in the form of HAsO_4_^2-^ and AsO_4_^3-^, and has less inclination to bind with the adsorbent [23]. In this regard, the diagram shows the reduction in the removal efficiency in alkaline conditions.

These trends can be claimed with the suggested mechanisms (Figure 6b) for the removal of arsenic using magnetic PES/ZVI beads at different pH values. At a pH < 4 and >6, protonation and a repulsion force are occurring, respectively. At a pH of 4–5, physical interactions, a chemical complexation, and electrostatic interactions are occurring, improving the removal efficiency. The complexation mechanism can occur between arsenic ions with oxygenate and sulfur functional groups in the backbone of PES by sharing ion pairs electrons. Furthermore, the distribution of ZVI on the surface of the adsorbent can serve as an electron donor which can enhance the removal efficiency by sorption or co-precipitation of arsenic ions. The formation of multilayers of corrosion products on the surface of ZVI by arsenic which has been adsorbed is well-reported [24]. In this regard, the existence of sulfur in the structure and iron nanoparticles render an excellent remediation of heavy metals via electrostatic interactions [24,25]. Accordingly, the solution was optimized to pH 5 for further experiments.

#### 3.2.3. Adsorbent Dosage

**Figure 7:**
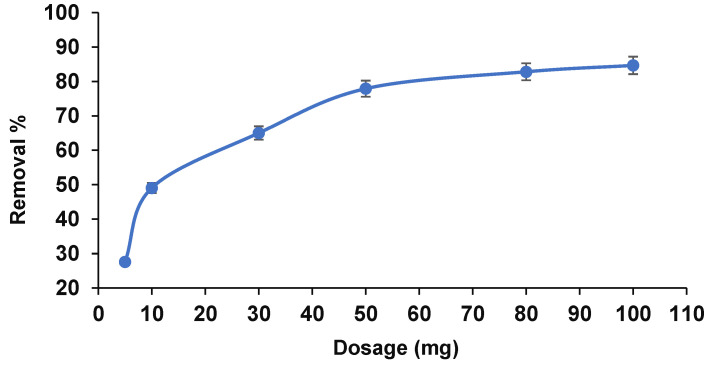
Effects of different amount of dosage on the removal efficiency (pH 5, 120 min reaction time, and 20 mg·L^−1^ arsenic).

To investigate the influence of adsorbent dosage on the treatment of arsenic from the soil, adsorption was accomplished with varying amounts of adsorbent from 5 to 100 mg under optimum conditions. Figure 7 displays a prompt increment from 27% to 78% with increasing the adsorbent dosage from 5 to 80 mg. This rapid increase was associated with a high number of active sites to bind with arsenic ions until equilibrium was gained at 80 mg of adsorbent. After that, the removal efficiency showed no significant change and became constant due to the occupation of all available sites by arsenic [25].

#### 3.2.4. Contact Time

**Figure 8:**
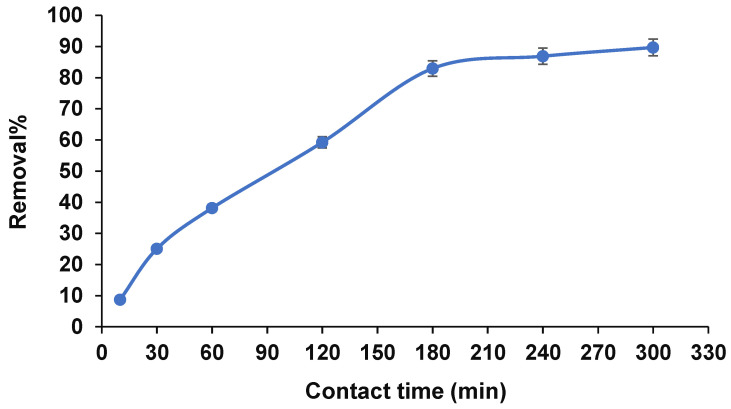
Effect of contact time on the removal efficiency (pH 5, 80 mg adsorbent dosage, and 20 mg·L^−1^ arsenic).

The adsorption/desorption rate of arsenic can control with the kinetics models. In this regard, contact time is a decisive factor in remediation. Figure 8 illustrates the effect of mixing time on adsorption efficiency. It observed that the prompt adsorption occurred within 10–180 min, then adsorption of arsenic maintained constant because equilibrium was achieved. Finally, to avoid re-adsorbed or re-precipitated arsenic, and considering experimental cost, 180 min was chosen for the following tests [26].

### 3.3. Kinetic Study

**Figure 9:**
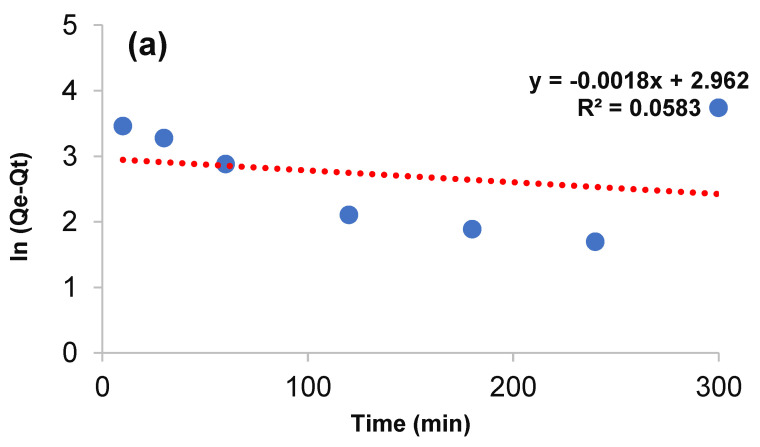

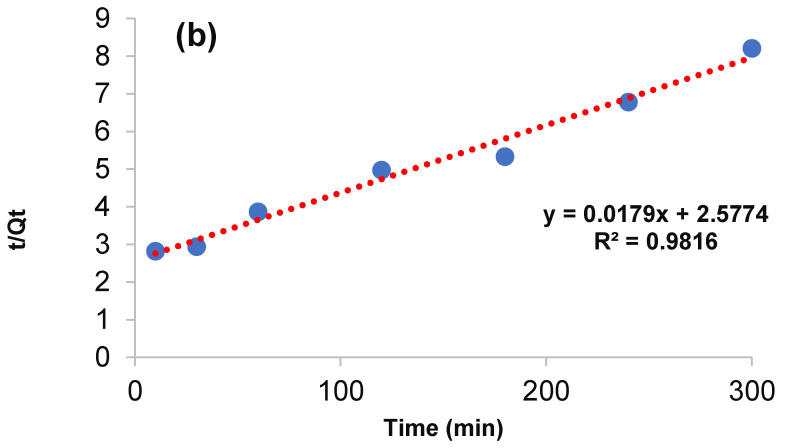
Linear form of (**a**) pseudo-first-order and (**b**) pseudo-second-order.

To understand the mechanism and rate of process, pseudo-first-order and pseudo-second-order were discussed. The pseudo-first-order is shown in Equation (3):

(3) $$\ln\left(Q_{e}-Q_{t}\right)=\ln Q_{e}-K_{1}t$$

where *Q_e_* (mg·g^−1^) and *Q_t_* (mg·g^−1^) are the amount of arsenic adsorbed at optimization condition. *K*_1_ (L·min^−1^) is the rate constant of the pseudo-first-order model. The values of *Q_e_* and *K*_1_ are obtained from the intercept and gradient of the line from the drawing of ln (*Q_e_* − *Q_t_*) versus *K*_1_t (Figure 9a). The pseudo-second-order model can be achieved by Equation (4):

(4) $$\frac{t}{Q_{t}}=\frac{1}{K_{2}Q_{e}^{2}}+\frac{t}{Q_{e}}$$

*Q_e_* (mg·g^−1^) and *Q_t_* (mg·g^−1^) are the same parameters described in Equation (4). *K_2_* (L·min^−1^), the constant rate of pseudo-second-order model, and *Q_e_* are gained through the gradient and intercept of the line from drawing *t*/*Q_t_* vs. *t* (Figure 9b). The parameters data are listed in Table 2, which indicating that the adsorption kinetic followed the pseudo-second-order model.

### 3.4. Equilibrium Adsorption and Isotherm Models

**Figure 10:**
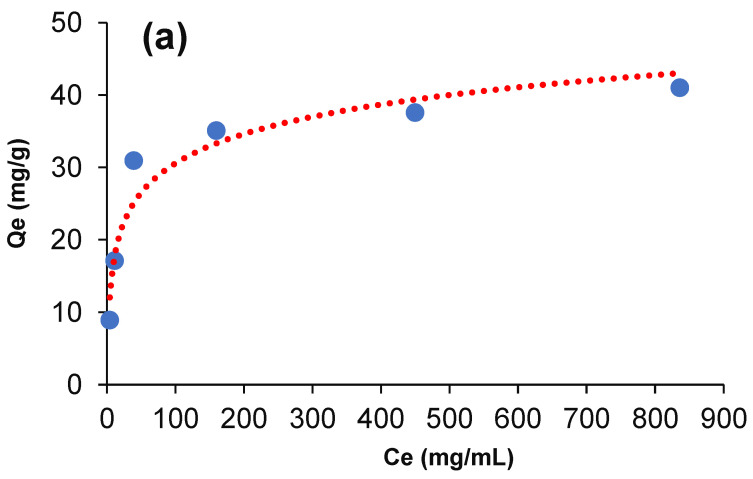

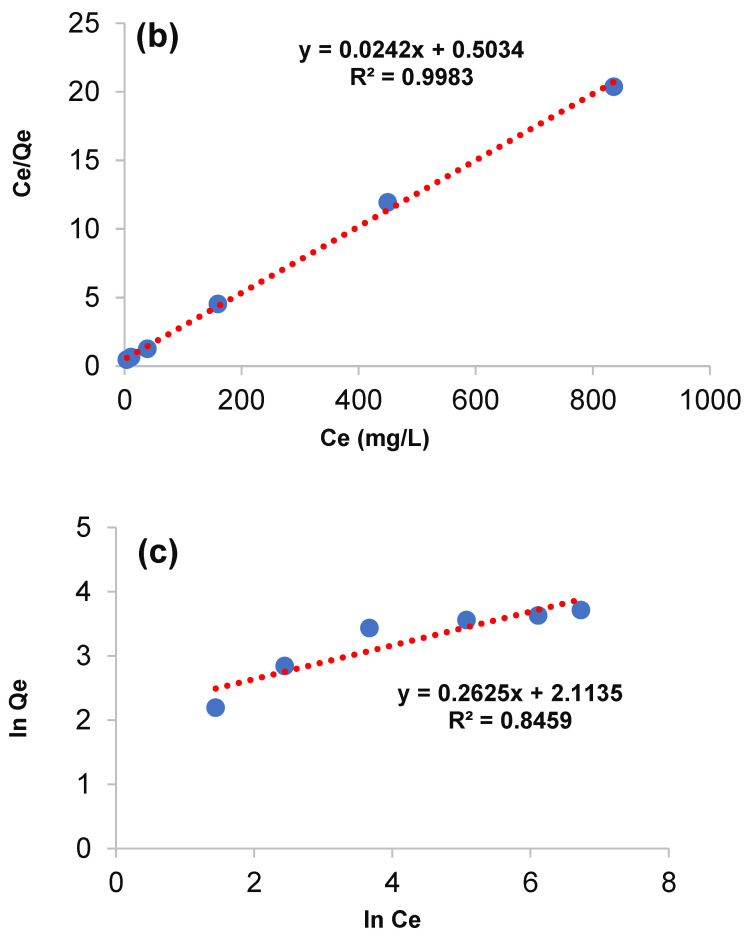
Isotherm models of (**a**) initial concentration and on equilibrium adsorption capacity (condition: pH 5, 80 mg adsorbent, and 20 mg·L^−1^ arsenic). Linear models of (**b**) Langmuir isotherm and (**c**) Freundlich isotherm models.

Adsorption isotherms were studied to achieve an adsorption pattern and a high tendency of metal ions with the adsorbent. The output of equilibrium adsorption isotherms is displayed in Figure 10a. As can be observed, the plot showed that the adsorption capacity was in the range of 8.94–41.02 mg·g^−1^ with an increase in the equilibrium concentration of arsenic ions from 40 to 1000 mg·g^−1^. The adsorption system reached equilibrium when the adsorption capacity was up to 35 mg·g^−1^, thus, with further increasing the initial concentration of arsenic, the sorption capacity of PES/ZVI was not improved more.

Furthermore, the Langmuir and Freundlich isotherm models were employed for the description of the mechanism of the adsorption process (Figure 10b,c). The values of the parameters are listed in Table 3. The obtained results indicate that the Langmuir model (Equation (5)) can be a better model to explain the adsorption process of arsenic on the PES/ZVI beads in comparison with the Freundlich model (Equation (6)), as expressed with the following equations:

(5) $$\frac{C_{e}}{Q_{e}}=\frac{C_{e}}{Q_{m}}+\frac{1}{K_{L}Q_{m}}$$

(6) $$lnQ_{e}=lnK_{F}+\frac{1}{n}lnC_{e}$$

where *Q_m_* (mg·g^−1^), *K_L_* (L/mg), *K_F_* [(mg·g^−1^)/(L/g)1/*n*], and *n* are the maximum adsorption capacity, Langmuir constant, Freundlich constant, and adsorption intensity, respectively. This notably stated that the pattern of adsorption of arsenic species onto PES/ZVI beads was sustained in a monolayer.

### 3.5. Temperature and Thermodynamic Studies

To investigate the thermodynamics, the adsorption process was assembled at three temperatures (293, 298, and 318 K). According to the results in Table 4, by the increasing temperature, the adsorption capacity was increased, indicating the endothermic process. This is because an increasing temperature leads to a significant change in the energy-dependent mechanism between the adsorbate and adsorbent. Enthalpy Δ*H*°, entropy Δ*S*°, and Gibbs free energy Δ*G*° are the thermodynamic parameters that were evaluated to describe the thermodynamic process of arsenic adsorption onto the PES/ZVI beads. These parameters were calculated as the following equations:

(7) $$ln\;Kc=\frac{\Delta S{}^{\circ}}{R}+\frac{\Delta H{}^{\circ}}{RT}$$

(8) ΔG°=−RT ln Kd

where *R* is the universal gas constant (0.008314 kJ·mol^−1^×K^−1^), *T* (K) is temperature, and *K_C_* = *Q_e_/C_e_* is the thermodynamic constant.

Based on the amount of Δ*G*°, the adsorption mechanism can be physisorption or chemisorption. If Δ*G*° is less than −20 kJ/mol, the mechanism can be considered physisorption, and when Δ*G*° is higher than −40 kJ/mol, it is the chemisorption mechanism. In this regard, arsenic adsorption onto the PES/ZVI beads can be ascribed to the physiochemical mechanism. Experimental data displays that an increment in temperature can lead to an increment in the adsorption capacity. Additionally, enthalpy was positive, thus, the adsorption process was endothermic.

### 3.6. Real Samples

For the evaluation of the performance of the adsorbent, various samples which contain a high concentration of arsenic were employed (Table 5). For this purpose, 80 mg of adsorbent was added to each soil, and solutions were stirred for 180 min under a pH of 5. The results showed that the efficiency of the adsorbent for the remediation of arsenic ions in the different soil was satisfied. Therefore, this adsorbent had a high potential for the extraction of arsenic ions even in a high concentration.

### 3.7. Regeneration Anc Recycle

**Figure 11:**
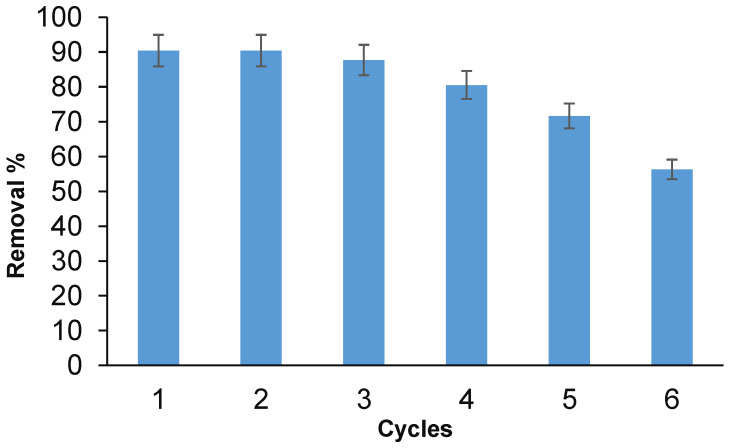
Recovery and recycle the adsorbent (pH 5, 80 mg adsorbent, and 20 mg/L arsenic).

The regeneration of PES/ZVI was studied with the continuous adsorption–desorption cycle of arsenic species. For the regeneration study, the arsenic ions were taken from the contaminated solution (20 mg·L^−1^) using 50 mg of adsorbent and 180 min of agitation time. Then, they were desorbed from the adsorbent using NaOH solution (0.1 M) for 10 min of shaking and they were then washed with excess DW. Figure 11 shows the adsorption–desorption cycles for six cycles. Based on the results, the removal efficiency decreased from 91% to 83% after four cycles and then decreased to 57% after six cycles. Thus, PES/ZVI was applicable for several continuous adsorption–desorption cycles of arsenic species.

### 3.8. Iron Leaching Test

**Figure 12:**
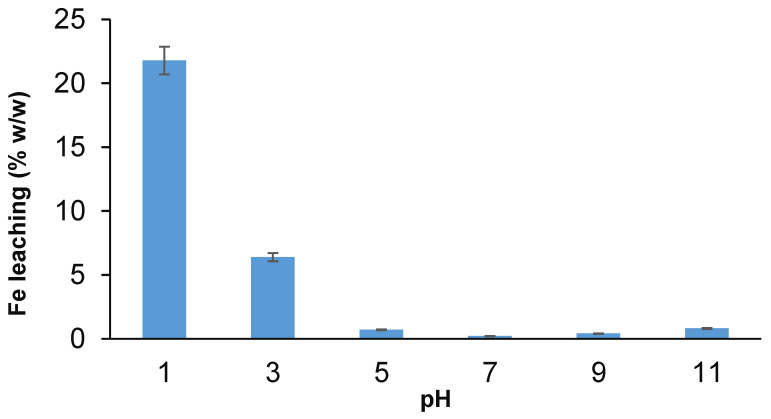
Leaching of iron (Fe) from microbeads at different pH values.

The leaching test is the main parameter in the removal study to claim the stability of the adsorbents. Thus, the chemical stability of the PES/ZVI composite was studied based on iron (Fe) leaching in the aqueous solution after 24 h with different pHs in the range of 1–11. Figure 12 shows that the leaching of Fe ions from PES/ZVI was highly influenced by the solution’s pH. As can be seen, the Fe ions were leached 23% at pH 1 and 7% at pH 3. While, at neutral pH and high pH values (9 & 11), the Fe ions leached <1%, which performed a good stability of the adsorbent at a pH > 7.

### 3.9. Comparison

The adsorption capacity of various adsorbents for the treatment of arsenic is reported in Table 6. As can be seen, the PES/ZVI nanocomposite has a significant adsorption capacity in comparison with other adsorbents. This result is attributed to the substantial porosity and high potential in binding with arsenic ions and eliminating them from the soil.

## 4. Conclusions

The object of this study was to investigate the function of PES/ZVI beads for the remediation of arsenic from soil. Various contributing parameters such as pH, initial concentration, dosage, and contact time that can exert influence on the functionality of adsorption have been studied. The structural characterization performed with FTIR, TGA, VSM, and SEM indicates that PES/ZVI beads have significant properties for the removal of arsenic from soil. According to the obtained data, it was portrayed that arsenic can be separated from soil by different mechanisms, such as adsorption and precipitation. In the synthesized adsorbent, iron nanoparticles had a decisive role to curb the adsorption process. Moreover, the kinetic parameters showed that a pseudo-second-order kinetic model was fitted with the experimental data. The thermodynamic model demonstrated that the adsorption process followed the physisorption mechanism. Additionally, the Langmuir isotherm justified the monolayer coverage of arsenic on the PES/ZVI beads.

## Figures and Tables

**Table 1:** Concentration of arsenic in different apatite-soils.

Sample	Original Concentration of Arsenic (mg·L^−1^)	Concentration of Arsenic in Apatite Treated Water at Different pHs (after Treatment)		
		pH = 3	pH = 6	pH = 10
G2	103.7	101.2	76.2	47.2
T1	147.3	142	118	63
GTSP	170.59	163	129	77
T15-20	156.67	147.11	124.8	82
Q3	87.7	86.9	62.4	32.7
Q8	72.32	72.14	53.7	29.11
Q12	146.2	139	123	67
Q16	134.5	130.4	99.4	60.5

**Table 2:** Kinetic parameters and values for arsenic ion uptake onto adsorbent.

Models	Parameters	Arsenic
Pseudo first order	*Q_e_* (mg·g^−1^)	18.916
	*K*_1_ (min^−1^)	0.0018
	*R* ^2^	0.0583
Pseudo second order	*Qe* (mg·g^−1^)	55.556
	*K*_2_ (g·mg^−1^·min^−1^)	0.000324
	*R* ^2^	0.981

**Table 3:** Parameters of isotherm arsenic ions on the adsorbent.

Metal	Langmuir Model			Freundlich Model		
	*Q_m_* (mg·g^−1^)	*K_L_* (L·mg^−1^)	*R* ^2^	1/*n*	*K_F_* (mg·L^−1^)^−1/*n*^	*R* ^2^
Arsenic	41.32	2.07	0.998	3.81	7.14	0.845

**Table 4:** Impact of temperature on sorption capacity and thermodynamic factors.

Adsorbate	T(K)	*Q_e_* (mg·g^−1^)	Δ*G* (kJ/mol)	Δ*H* (kJ/mol·K)	Δ*S* (kJ/mol·K)
arsenic	20	34.45	−0.76	28.31	0.097
	25	35.77	−1.45		
	45	38.03	−3.31		

**Table 5:** The measurement of arsenic in different samples.

Samples	Native Arsenic (ppb)	*C*_0_ (after Acidic Treatment)	*C_e_* (after Removal)	R%
G2	103.7	101.2	19	81.22
GTSP	147.3	142	28	80.28
T1	170.59	163	45	72.39
T15-20	156.67	147.11	39	73.48
Q3	87.7	86.9	11.8	86.42
Q8	72.32	72.4	8.1	88.81
Q12	146.2	139	33	76.25
Q16	134.5	130.4	29.4	77.45

**Table 6:** The comparison of different adsorbents for the separation of arsenic.

Adsorbent	Adsorbate	*Q_m_* (mg·g^−1^)	Ref.
Biochar/AlOOH	Arsenic	17.41	[27]
Magnetic oxide biochar	Arsenic	14.36	[28]
Fe_2_O_3_ Al_2_O_3_	Arsenic	0.66 0.17	[29]
Copper (II) oxide	Arsenic	1.086	[30]
Zeolite-based/ZVI	Arsenic	38.26	[31]
PES/ZVI	Arsenic	41.32	This study

## Data Availability

Not applicable.
